# Editorial: Natural products and microbial interactions: from antimicrobial activity to microbial modulation

**DOI:** 10.3389/frabi.2026.1901611

**Published:** 2026-06-22

**Authors:** Pablo Mendez-Pfeiffer, Manuel G. Ballesteros-Monrreal, Mayra Mendez-Encinas, Bryan Ortiz, Yaxsier De Armas

**Affiliations:** 1 Centro de Nanociencias y Nanotecnología, Universidad Nacional Autónoma de México, Ensenada, Baja California, Mexico; 2 Departamento de Ciencias Químico Biológicas y Agropecuarias, Universidad de Sonora, Caborca, Sonora, Mexico; 3 Instituto de Investigaciones en Microbiología, Facultad de Ciencias, Universidad Nacional Autónoma de Honduras, Tegucigalpa, Honduras; 4 Centro Universitario de Ciencias de la Salud, Universidad de Guadalajara, Guadalajara, Jalisco, Mexico

**Keywords:** antibacterial activity, antimicrobial activity, biofilm, microbial modulation, natural products

The growing burden of antimicrobial resistance has led to a search for new therapeutic alternatives to conventional antimicrobial therapies, increasing interest in natural products as a source of biologically active compounds (AlSheikh et al., 2020). These products derived from plants, fungi, and microorganisms, contain a variety of structurally diverse molecules capable of interacting with microbial cells through multiple mechanisms. Although the development of new antimicrobials has traditionally focused on inhibiting growth or eliminating the pathogen, this approach does not always control key processes involved in pathogenesis and may favor the selection of resistant strains (Alum et al., 2025a; Pérez-Flores et al., 2025). The exploration of bioactive natural products represents a promising strategy to address infections caused by diverse pathogens, not only because of their direct antimicrobial potential, but also because of their ability to modulate essential processes of microbial virulence. This approach enables a shift from a perspective centered exclusively on growth inhibition toward broader anti-infective strategies aimed at reducing pathogenicity, interfering with biofilm formation, disrupting microbial communication, and improving therapeutic responses against resistant or persistent microorganisms (Fernandes et al., 2025; Mulat et al., 2025; SeyedAlinaghi et al., 2025).

This Research Topic, “*Natural products and microbial interactions: from antimicrobial activity to microbial modulation*,” was conceived as a platform to broaden the perspective on natural products as microbial modulators in complex environments. The five articles included in this Research Topic illustrate progress in the evaluation of natural products not only as antimicrobial agents, but also as modulators of microbial behavior, persistence, community balance, and host-associated responses. These articles demonstrate that the study of natural products goes beyond planktonic microbial inhibition.

Gutierrez-Montiel et al. evaluated extracts of *Psidium guajava* against clinically relevant bacteria associated with healthcare environments. The authors examined the effect of the extracts on the growth of *Enterococcus faecalis* and *Staphylococcus epidermidis*, as well as their effect on adhesion, biofilm formation, and extracellular matrix composition. Their findings showed that the effect depends on the bacterial strain evaluated and the formulation of the extract. It also demonstrates that new strategies, such as microencapsulation, can improve the efficacy of these natural products at lower concentrations, while also emphasizing the need to consider their potential cytotoxic effects during formulation development.

On the other hand, the study conducted by Mundanchira et al. evaluated a complex botanical formulation against biofilms formed by Gram-negative bacteria, Gram-positive bacteria, and *Candida albicans*. The results showed that antimicrobial efficacy depended on microorganism, the concentration evaluated, and the conditions of exposure, showing that these natural products can serve as broad-spectrum antimicrobial agents.

Biofilm formation represents a central matter across the studies included in this Research Topic. These structures, composed of microbial communities, are dynamic systems in which cells within an exopolysaccharide matrix communicate by modulating their gene expression, metabolism, and stress responses, resulting in increased antimicrobial resistance and persistence. The ability of natural products to interfere with the development of these biofilms, their architecture, and survival may be as relevant as their effects on planktonic microorganisms.

This is reflected in the review published by Sahu et al., which highlights advances in the evaluation of the activity of natural products, such as flavonoids, terpenoids, and phenolic compounds, against *Candida glabrata*. Here, it is proposed that these natural products exert their effect through various mechanisms, such as the destruction of cell membranes, ergosterol inhibition, oxidative stress, modulation of efflux pumps, suppression of virulence, and a decrease in biofilm production. The review also emphasizes the potential use of natural products as adjunctive agents capable of enhancing the efficacy of conventional antifungal therapies.

Debta et al. expand the definition of natural products beyond plant-derived compounds. The authors examine metabolites produced by probiotic microorganisms as potential tools for targeting fungal biofilms and persister cells. These cells are phenotypically tolerant subpopulations that can survive lethal antimicrobial exposure and contribute to recurrent infection. By discussing postbiotic metabolites, this review introduces a form of microbial modulation in which beneficial microorganisms, or their products, may interfere with adhesion, virulence, quorum sensing, matrix production, and survival of pathogenic fungi. This perspective is especially relevant because it shifts attention from microbial eradication alone toward the manipulation of microbial interactions.

Finally, Rath et al. presents a broader ecological perspective, in which the microorganism-host interactions are closely associated with the use of these natural products. Gingivitis is associated with the accumulation of biofilms in the oral cavity, dysbiosis of the normal microbiota, and the host’s inflammatory response. This review describes the use of natural products with anti-inflammatory, antioxidant, tissue-regenerating, and microbiota-modulating activity. At the same time, it highlights one of the principal challenges in the therapeutic application of natural products, namely the lack of standardized formulations and the high variability in the concentrations of bioactive compounds.

Collectively, the articles in this Research Topic demonstrate that natural products can influence microorganisms at multiple biological levels. They may inhibit growth, reduce adhesion, disrupt biofilm matrices, interfere with resistance mechanisms, alter microbial signaling, or modulate the relationship between microbial communities and the host. However, this Research Topic also underscores important challenges, including variability in chemical composition, limitations in formulation, the lack of standardized antimicrobial evaluation methods, potential cytotoxicity, and the scarcity of *in vivo* and clinical evidence.

Future research should prioritize the chemical characterization and standardization of natural preparations, the evaluation of biologically relevant biofilm and microbial community models, and the development of formulations that improve stability, bioavailability, and targeted delivery. Greater emphasis should also be placed on methodologies that clarify how natural products interact with microorganisms and how they influence virulence, pathogenesis, antimicrobial resistance, and host–microbe interactions. Such efforts will be essential to translate the promising biological properties of natural products into safe, reproducible, and clinically meaningful applications (Wang et al., 2023; Alum et al., 2025b; Farah et al., 2026).
